# European policies for public health in border regions: no European mindset as yet

**DOI:** 10.1186/s12889-024-18175-9

**Published:** 2024-03-08

**Authors:** Brigitte A.M. van der Zanden, Christian J.P.A. Hoebe, Klasien Horstman

**Affiliations:** 1 Foundation euPrevent, Het Overloon 2, Heerlen, 6411TE The Netherlands; 2 Department of Social Medicine, Care and Public Health Research Institute (CAPHRI), Faculty of Health, Medicine and Life Sciences, Maastricht University, P.O. box 616, Maastricht, 6200 MD The Netherlands; 3 Department of Sexual Health, Infectious Diseases and Environmental Health, Living Lab Public Health Mosa, South Limburg Public Health Service, P.O. box 33, Heerlen, 6400 AA The Netherlands; 4 Department of Medical Microbiology, Infectious Diseases and Infection Prevention, Care and Public Health Research Institute (CAPHRI), Faculty of Health, Medicine and Life Sciences, Maastricht University Medical Centre (MUMC+), P.O. box 5800, Maastricht, 6202 AZ The Netherlands; 5 Department of Health, Ethics and Society, Care and Public Health Research Institute (CAPHRI), Faculty of Health, Medicine and Life Sciences, Maastricht University, P.O. box 616, Maastricht, 6200 MD The Netherlands

**Keywords:** European Union, Narrative policy analysis, Public health, Prevention, Health promotion, European knowledge structure, Border regions, Cross-border

## Abstract

**Background:**

The sudden emergence of COVID-19 in 2020 demonstrated that Europe was not prepared for a public health crisis like this pandemic. In the European Union, matters of health have remained primarily under the jurisdiction of individual Member States. However, certain events, such as the Kohll-Decker ruling on free mobility of health services and the COVID-19 pandemic, compelled the EU to address health matters in border regions. This study examines how EU policies address public health in border regions. To that end, we have drawn from border studies, a field that provides insight into the fluidity and complexity of borders in everyday life. Besides that we used constructivist policy studies as a lens for the analysis of EU policy documents.

**Methods:**

A policy discourse analysis was conducted to explore how European policy addresses the development of a transnational, European public health in border regions. Key European policy documents published between 2002 and 2027 were analysed to understand how policies are constructed and problems are framed. The analysis was guided by research questions and the theoretical approach.

**Results:**

The analysis reveals that, while having limited competences in the field of health care, the EU is slowly developing a rationale and a knowledge base to increase its competences in health care. It also shows that in the field of public health, the EU argues for addressing health determinants and promoting healthy lifestyles, though it does not address health promotion in border regions. The EU’s authority in public health in border regions revolves primarily around addressing physical, biological and chemical threats rather than social health problems.

**Conclusion:**

Though the EU has carefully developed a transnational perspective on health care, the EU has not developed any authority with respect to transnational public health. Though public health and health promotion in border regions have been confronted with specific challenges, neither specific Member States nor the EU have a transnational collaborative perspective that does justice to the characteristics of border regions. When it comes to public health in border regions, there is no European mindset as yet.

## Introduction

The diverse responses to the sudden and unexpected emergence of COVID-19 in 2020 demonstrated that Europe was not prepared for a pandemic. To control the pandemic, many states introduced national measures, such as wearing a facemask, social distancing, limiting group sizes, reducing travel, testing and vaccination strategies. Some countries also decided to close their borders to reduce cross-border mobility: by physically reinstating the formal state borders, countries reduced cross-border mobility in an unsuccessful attempt to keep the virus outside.

These national policies did not take into account that 30% of the EU population lives in so-called border regions [1–3]. Since the European Union has embraced the ‘free movements of persons’ in accordance with the Schengen Agreement of 1985 and the Schengen Convention of 1990 [4] and the EU principle of the free movement of goods and services [5, 6], intense cross-border mobility– for work, leisure, shopping, family visits, care and education– has become a self-evident aspect of daily life in border regions. From that perspective, it is not surprising that border closures were not particularly successful [7], especially because the every-day mobility necessary in border regions required too many exemptions. However, these responses reflect a remarkable national reflex in relation to European public health and, as such, they raise the question as to what extent– and how– European policies for public health take border regions into account.

In this article, we explore this question by analysing how transnational European public health in border regions is addressed in European policy discourses. In the next section, we first introduce how Europe has dealt with the potential European character of public health to date. Next, we sketch the theoretical background of the study and present the methodology. After having presented the results of the policy analysis, we discuss these findings within the context of relevant literature.

### Health as a European issue?

While Europe has developed as an open market and has embraced the free movement of persons, goods and services in the EU, the same cannot be said in relation to health. In the last consolidated version of the Treaty on the Functioning of the European Union [6], of which the first version appeared in 2002 [5], the EU formally stated that health care is a matter for Member States. This entails that the Member States agreed that the EU’s role with respect to health does not go beyond supporting, coordinating, and complementing national policies [6]. However, in 1998 the EU was forced to stretch this policy to a degree.

In 1998, the European Court of Justice ruled that the free movement of goods and services within Europa also applies to health care, the so-called Kohll-Decker Ruling [8, 9]. Interestingly, this ruling was the result of an initiative from citizens. In the early 1990s, Mr Kohll and Mr Decker, both living in Luxembourg, decided to go abroad for, respectively, prescribed glasses and dental care. Both submitted their expenses to their health insurance companies for reimbursement, but both received a rejection. The insurance companies argued that there was no medical emergency or need for them to seek help in a neighbouring country or another EU Member State. Mr Kohll and Mr Decker went all the way to the European Court of Justice (ECJ) to fight this decision. The Member States found this quite problematic and nine of the - at that time − 15 EU Member States voiced their concerns to the ECJ, warning the ECJ about the impact if it were to rule in favour of Mr Kohll and Mr Decker. This had no effect and in 1998 the European Court of Justice supported the arguments of Kohll and Decker and ruled that the free movement of goods and services also applies to health care. This forced the EU to consider how to respond. In 2011, after a long and extensive negotiation period between the EU and the Member States, the EU responded by establishing the patient rights directive. This directive obliges EU Member States to translate into national legislation the right of patients to search for health care in Member States other than their home country. However, though this goal must be met by all Member States, in line with the idea that the role of the EU with respect to health matters should be limited, Member States were not told how they should implement this directive. In 2021 and 2022 two reports were published by the EU which described in detail the challenges of implementing the directive [10, 11].

A more recent example of the EU’s interference in health matters was evident during the COVID-19 pandemic, namely the introduction of the digital COVID-19 passport. Since the start of the COVID-19 pandemic, the EU worked with its Member States to strengthen Member States’ health systems in order to limit the spread of the virus. To this end, the EU coordinated action at EU level and recommended public health measures to EU Member States, such as the introduction of the EU digital COVID-19 passport which allowed EU citizens to move more easily within the EU. Compared with the Kohll-Decker ruling, the EU COVID-19 policy was not particularly controversial among policymakers and did not meet extensive criticism from EU Member States. The COVID-19 crisis and the impact of pandemic control measures were considered urgent problems that blocked the free movement of goods and persons, and the COVID-19 passport was considered an adequate response as it enabled the resumption of cross-border mobility. Apart from this, as a too, the COVID-19 passport matched existing national public health infrastructures while not competing with them.

These examples demonstrate that in specific situations the EU will go beyond its limited role when it comes to health matters. The new directive legitimises the EU holding countries accountable for ensuring that their citizens can fulfil their rights with respect to the free movement of services and goods in the field of health care, a right that may be of extra importance in border regions. Prevention and health promotion encounter various obstacles in border regions. For example, the introduction of sugar tax in Norway (not an EU Member) to decrease sugar intake stimulated people to buy their sweets in Sweden [12], and in the Netherlands raising the age limit for buying alcohol from 16 to 18 resulted in young people taking their custom to Belgium and Germany. Both examples illustrate the so-called waterbed effects of policies in border regions. If specific prevention and health promotion policies in one country do not align with those in a neighbouring country, this may decrease the legitimacy and the efficacy of these policies. In numerous publications, the World Health Organisation (WHO) has called for more attention should be paid to the effects of marketing in border regions in relation to alcohol and tobacco. This raises the question as to how public health in border regions is addressed in EU policies.

### Theoretical background

For our study, we draw from two different fields, namely border studies and constructivist policy studies. While border studies helps to reflect on the notion of borders and border regions, constructivist policy studies offer ideas for an in-depth understanding of how policies shape the world [13–16].

In border studies many scholars have argued that borders should be considered as cultural, economic, political and social constructs. Aure [13], for example, explained that to understand life in border regions, formal, national and political borders are far less important than everyday cross-border social-economic relations. Kolossov [17] argues that, in practice, borders are rather fluid. In the same vein, according to Walters [15], the EU is characterised by ‘multiple, fluid spaces of regions, markets and cities connected by networks of communication and transport and traversed by flows of goods, people, information and capital’ [15] (p. 676). While a geopolitical perspective on borders as demarcating nation states is dominant in the domains of policy and law, scholars such as Kolossov [17], Aure [13] and Walters [15, 18] argue that developing adequate policies requires focusing on the fluidity of borders in everyday life. Studies of life in border regions also indicate that living with different governance regimes close at hand provides flexibility and freedom: if parents do not like the national educational system, they can put their children in schools in the neighbouring country, and if some groceries are very expensive in the home country, it is easy to shop across the border.

To analyse EU policies of public health in border regions, we draw from constructivist policy studies. Scholars in this field e.g. Borras and Edler, have stressed that governance - instead of steering - is a process of co-construction by state and diverse non-state actors, and the dynamics of co-construction is affected by a variety of social, cultural, economic and scientific-technical processes [19]. More specifically, for our question about transnational public health - a scientific professional field - we use the work of Barry [20–22]. According to Barry, transnational governance is related to the emergence of transnational knowledge dynamics such as controversies about climate change [21]. Although controversies are often seen as obstacles to transnational collaboration and consensus, Barry argues that these controversies stimulate innovations in governance, such as the development of transnational technological standards and metrics. Attempts to develop transnational mechanisms to tackle the problem of climate change were accompanied by disagreements about, for instance, the extent of the climate problem, its urgency, the causes and consequences, and the reliability of measuring instruments, models and data that stimulated new scientific-technological strategies. These controversies are the machinery that shape transnational governance. So, according to Barry, to get insight into a transnational policy landscape, it is important to get insight into transnational knowledge dynamics. Kingdon [23] demonstrated the inevitably selective character of policy processes: some problems, objects or issues receive more attention than others in the prioritization of problems and in these processes, so-called ‘hidden participants’ such as scientists, consultants and analysts play a major role. However, their role in putting some problem definitions and solutions forward depends on a ‘window of opportunity’: a moment where– often by coincidence– a specific problem definition or solution become embraced by many stakeholders and is rapidly developed. This process is influenced by what Kingdom calls ‘social entrepreneurs’: people who– on behalf of organisations, institutes or stakeholders– spend time and resources on lobbying for the issues and policies they represent. If their work is not successful and topics are not put on the agenda, Kingdon interprets this in terms of the lack of a ‘window of opportunity’.

The core of Bacchi’s [24–27] critical policy analysis focuses not on policy construction through the agenda-setting dynamics of state and non-state actors, but on the representation of problems. She argues that problems are often presented as natural, objective facts, while they should be analysed as specific representations that are loaded with normative assumptions and have normative consequences. An example, according to Bacchi [26], is policies that aim to increase the number of women in influential positions by educating these women. While this may seem ‘logical’, a critical policy analysis may, for instance, indicate that such an educational strategy defines lack of education of women as the main problem rather than the power dynamics of old boys’ networks. In line with this, women are expected to improve their education, and gendered organisation patterns remain in place. While Kingdon [23] stresses that we need to study how problems acquire status and are prioritized on a government’s agenda, Bacchi [24–27] recommends critically analysing how these ‘problems’ are constructed as ‘problems’ of a particular kind and to critically reflect on the normative assumptions in policies.

## Methods

To explore the question ‘How do European policies address and construct public health in border regions?’, we conducted a constructivist policy analysis. Although we consider policy and governance as processes in which many institutional and cultural actors play a role, we analysed EU policy documents in order to understand the construction of policies and how problems and solutions are framed in the process [28, 29]. The analysis of policy documents allows us to map how specific problem definitions have developed. According to Asdal [28], editing and changing the presentation of content is part of transforming reality. For example, she shows how a letter from a veterinarian in Norway describing a problem relating to an aluminium factory and the effects of the factory’s emissions have on local livestock, resulted in - through all kinds of modifications - to policy at a national level. The issue that was raised in the letter quickly changed from an exemplary situation to a general situation, from an emotional argument to a factual argument, and from an individual and local issue to a national issue. The final report has become rather detached from the initial context. In other words, texts, and networks of texts play an important role in constructing the world.

**Fig. 1:**
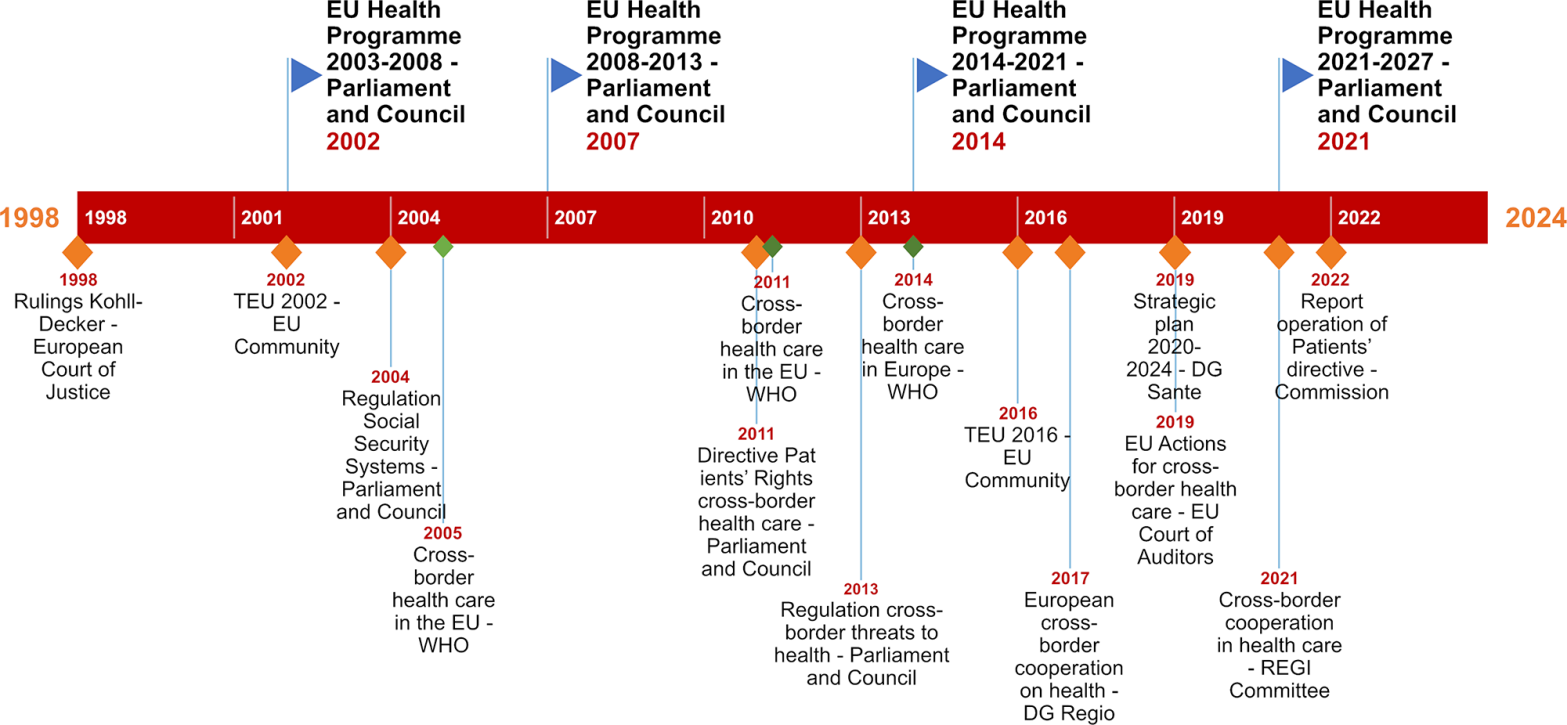
Timeline of EU and WHO policy documents used for analysis (blue triangles: main documents in analysis; orange rhombus: all EU documents selected for this paper and green rhombus: related WHO documents). Full name of documents in Table 1

Inspired by the work of Asdal [28], we analyse whether and how EU policies on public health consider public health in border regions. For that purpose, we selected key EU policy documents that were digitally available on the website of the European Commission, supplemented by regulations, directives, guidelines, programmes, strategic plans and studies. We carried out the selection in four steps. First, we selected all policy documents issued by the EU and its departments from 2002 until May 2022 by using the key words ‘cross-border’, ‘(public) health’ and ‘Europe’. We started in 2002 because in that year the first integrated Community programme for public health and health promotion was adopted. This step resulted in a list of 16 documents (see Fig. 1, points on the timeline are marked orange and blue).

**Table 1:** Legend of Fig. 1 (‘Name in Fig. 1’- the name as it is mentioned in Fig. 1, ‘Full name of document and reference’– the full name of the document and its reference number’)

**Blue triangle in Fig.** 1**: documents analysed in this article**	
**Name in Fig.** 1	**Full name of document and reference**
2002	Decision No 1786/2002/EC of the European Parliament and of the Council of 23 September 2002 adopting a programme of Community action in the field of public health (2003–2008) - Commission Statements [30]
EU Health Programme 2003–2008– Parliament and Council	
2007	Decision No 1350/2007/EC of the European Parliament and of the Council of 23 October 2007 adopting a programme of Community action in the field of public health (2008–2013) [31]
EU Health Programme 2008–2013– Parliament and Council	
2014	Regulation (EU) No 282/2014 of the European Parliament and of the Council of 11 March 2014 on the establishment of a third Programme for the Union’s action in the field of health (2014–2020) and repealing Decision No 1350/2007/EC [32]
EU Health Programme 2014–2021– Parliament and Council	
2021	Regulation (EU) 2021/522 of the European Parliament and of the Council of 24 March 2021 establishing a Programme for the Union’s action in the field of health (‘EU4Health Programme’) for the period 2021–2027, and repealing Regulation (EU) No 282/2014 [33]
EU Health Programme 2021–2027– Parliament and Council	
**Orange rhombus in** Fig. 1: **EU related documents**	
**Name in** Fig. 1	**Full name of document and reference**
1998	Raymond Kohll versus Union des Caisse de Maladie [8].
Rulings Kohll-Decker– European Court of Justice	
1998	Nicolas Decker versus Caisse de Maladie des Employés Privés [9].
Rulings Kohll-Decker– European Court of Justice	
2002	Consolidated versions of the Treaty on European Union and of the Treaty establishing the European Community [5].
TEU 2002– EU Community	
2004	Regulation (ec) no 883/2004 of the European Parliament and of the Council of 29 April 2004 on the coordination of social security systems [34]
Regulation on social Security Systems - Parliament and Council	
2011	Directive 2011/24/EU of the European Parliament and of the Council of 9 March 2011 on the application of patients’ rights in cross-border health care [35]
Directive patients’ Rights cross-border health care - Parliament and Council	
2016	Consolidated versions of the Treaty on European Union and of the Treaty on the functioning of the European Union. [6]
TEU 2016– EU Community	
2017	European cross-border cooperation on health: theory and practice [36]
European cross-border cooperation on health - DG Regio	
2019	Strategic Plan 2020–2024 DG Health and Food Safety (SANTE) [37]
Strategic plan 2020–2024 - DG Sante	
2019	EU actions for cross-border healthcare: significant ambitions but improved management required. Special report No 07, 2019 [38]
EU Actions for cross-border health care - EU Court of Auditors	
2021	Research for REGI Committee– Cross-border cooperation in health care [39]
Cross-border cooperation in health care - REGI Committee	
2022	Commission Report on the operation of Directive 2011/24/EU on the application of patients’ rights in cross-border health care [40]
Report operation of Patients’ directive - Commission	
**Green rhombus in** Fig. 1: **WHO-related documents**	
**Name in** Fig. 1	**Full name of document and reference**
2005	Cross-border health care in Europe [41]
Cross-border health care in the EU - WHO	
2011	Cross-border health care in the European Union: mapping and analysing practices and policies [42]
Cross-border health care in the EU - WHO	
2014	Cross-border health care in Europe [43]
Cross-border health care in Europe - WHO	

Second, after a first tentative reading of the EU documents, we decided to also include documents of the WHO related to the three key words of ’cross-border’, ‘(public) health’ and ‘Europe’. As EU policy documents often refer to the WHO and this organisation is presented as an important knowledge institute for public health, we expected the WHO to play an important role in defining cross-border public health in Europe. Therefore, we also selected relevant WHO documents. These WHO documents are marked green on the timeline presented in Fig. 1.

Third, we selected the four policy documents that are most relevant to grasp how EU public health policies deal with health issues in border regions in the sense that these have been enshrined in formal EU decisions and regulations by the European Parliament and the Council, as listed below:

1. Decision No 1786/2002/EC of the European Parliament and of the Council of 23 September 2002, adopting a programme of Community action in the field of public health (2003–2008) [30];
2. Decision No 1350/2007/EC of the European Parliament and of the Council of 23 October 2007, establishing a second programme of Community action in the field of health (2008–2013) [31];
3. Regulation (EU) No 282/2014 of the European Parliament and of the Council of 11 March 2014 on the establishment of a third Programme for the Union’s action in the field of health (2014–2020) and repealing Decision No 1350/2007/EC [32]; and.
4. Regulation (EU) 2021/522 of the European Parliament and of the Council of 24 March 2021 establishing a Programme for the Union’s action in the field of health (‘EU4Health Programme’) for the period 2021–2027, and repealing Regulation (EU) No 282/2014 [33].

Finally, in order to validate our selection process, we presented this sample and selection criteria to three people closely involved in health policy processes in the Brussels arena and working for DG Sante and Euregha, and to get feedback on the quality of the procedure and to check for relevant and possibly missed documents. Based on their feedback, the sample was not changed.

These policy documents reflect existing discourses, initiatives and urgencies in the framework of European public health policy and have shaped EU public health policy. In these four policy documents, references are made to more general health policy documents of the EU (Commission, Parliament and European Court of Justice), as well as to the WHO (in the timeline in Fig. 1 marked in orange and green). As these referenced documents provide the context in which public health policy emerged, they are included in the analysis.

The analysis of the documents was guided mostly by the research question and the theoretical approaches and partly inductively. The analysis was done on paper and in Excel by two researchers, namely BZ and KH, and consisted of several steps. We started by the double reading of each policy document by two researchers. Next, with the research question and theoretical background in mind, we identified core themes and questions. First, where and how were the notions health care and public health, prevention, health promotion discussed in the papers? Second, inspired by border studies, we identified such expressions as cross-border and transnational governance/actors/institutions. Third, informed by the work of Barry, we looked at how institutions and strategies of science and technology were put forward. Fourth, inspired by Kingdon, we looked at indications of the dynamics and the rationale of agenda-setting in the policy papers. Finally, making use of Bacchi’s work, we looked at the definition of problems and solutions in policy documents.

Third, we re-read each document and selected text fragments associated with the chosen questions and themes. These were broad fragments, as we also included the context in which these themes were addressed. These fragments were then placed in an excel file. Fourth, we familiarised ourselves even more with the fragments and looked for chronological and systematic patterns of links between these themes. We specifically compared expressions linked with health care and public health. This resulted in a specific narrative about the attention for transnational and cross- border issues in public health in EU policy documents.

In the next section, we present the results of the analysis. The answers to the question if and how different health practices– health care, public health, prevention, health promotion - are considered from a transnational perspective, and to what extent border regions are taken into account, form the basis of the three result sections.

## Results

In the following sections we present how European policy discourse reflects a European, transnational perspective with respect to health care and public health and how cross-border public health issues are framed in these policy documents.

### EU policies for health care: constructing a transnational knowledge strategy and limited authority

While it is generally acknowledged that the role of the EU in health matters is limited to supporting, coordinating and complementing national policies, it is interesting to note that, nevertheless, the EU is slowly developing a rationale and a knowledge base for increasing its authority in matters of health.

First, European policy documents are increasingly articulating and calling attention to transnational health issues that cannot successfully be dealt with by national states. On several occasions, within the context of discussions about, for example, rare diseases and COVID-19, the European Parliament stresses the limits of national states in achieving health aims. These problems are considered as transnational by nature and as too complex to be controlled by individual nation states [30]. (p. 271/4, no. 22)

In the 2007 EU policy document, the Commission argues again that:

Since the objectives of this Decision cannot be sufficiently achieved by the Member States due to the transnational nature of the issues involved, and can therefore, by reason of the potential for Community action to be more efficient and effective than national action alone in protecting the health and safety of citizens, be better achieved at Community level [31]. (p. 301/7, no. 37)

The limitations of national policies in dealing with urgent health threats became very clear with the outbreak of the COVID-19 pandemic. Not surprisingly, in the 2021–2027 EU policy plan on health, one of the main themes is COVID-19.

Second, in line with the increasing attention to the transnational character of health matters, the policy analysis reveals that the EU has invested in a European knowledge base with respect to curative health care. While Member States rely on their own national health knowledge infrastructures, the EU has relied, to a large degree, on global policy-knowledge bodies such as the WHO. The 2002 and 2007 policy documents, for example, refer to cooperation with the WHO and the Organisation for Economic Co-operation and Development (OECD):

Article 11, International cooperation: In the course of implementing the programme, cooperation with third countries and with international organisations competent in the sphere of public health, in particular the World Health Organisation, the Council of Europe and the Organisation for Economic Cooperation and Development, or able to have an impact on public health […] shall be encouraged in accordance with the procedure laid down in Article 9(3). In particular, the health information system and the capacity to respond to health threats should be, where appropriate and possible, coordinated with the activities of the World Health Organisation [30]. (p. 271/8)

Article 12, International cooperation: In the course of implementing the Programme, relations and cooperation with third countries that are not participating in the Programme and relevant international organisations, in particular the WHO, shall be encouraged [31]. (p. 301/10)

However, since 2007 the EU has increasingly referred to European institutes, such as the European Centre for Disease Prevention and Control (ECDC), which focuses on the control of infectious diseases, and the European Medicines Agency (EMA), which deals with EU admission and the safety of medicines. These institutes articulate notions of transnational collaboration and transnational knowledge and were established either by the European Parliament via a regulation or by a decentralised agency of the European Union. The 2007 EU policy document states:

For the attainment of the objectives of the Programme, the Commission shall, in close cooperation with the Member States: (b) ensure the necessary cooperation and communication with the European Centre for Disease Prevention and Control and other relevant EU agencies in order to optimise the use of Community funds [31]. (p. 301/8)

The 2021 EU policy document again underlined the importance of the ECDC:

… in synergy with other Union instruments, programmes and funds, without prejudice to Member State competences and in close cooperation with the ECDC [33]. (p. 107/13)

While the importance of ECDC is further supported by the communication of the Commission of 11 November 2020 entitled ‘Building a European Health Union: Reinforcing the EU’s resilience for cross-border health threats’ [33]. (p.107/3)

Third, the Directive on patients’ rights in cross-border health care [35] allows the EU to develop transnational vehicles to stimulate Member States to act in this respect. For that purpose, in 2013 the EU introduced the so-called European reference networks (ERNs), a (virtual) network for knowledge institutes and healthcare providers from all over Europe to ‘*increase access to medical expertise and information for specific conditions beyond national borders*’ [32] (p. 86/7). The networks that were actually established centre around rare diseases [35] (p. 88/61, article 8), a choice that indicates the EU’s careful navigation in this field. While health issues are considered a national issue, EU interference concerning rare diseases is not really controversial, as all Member States have only few cases and an obvious need to share knowledge and resources about these diseases and about the highly specialised treatment. The EU has anticipated an increase in these networks and has been willing to support Member States in participating in these networks:

… through the increase in the number of European reference networks established in accordance with Directive 2011/24/EU of the European Parliament and of the Council (1) (“European reference networks”), the increase in the number of healthcare providers and centres of expertise joining European reference networks, and the increase in the number of Member States using the tools developed [32]. (p. 86/7)

The Programme should contribute to the upscaling of networking through ERNs and other transnational networks [33]. (p. 107/7, no. 33)

In the 2021 EU policy document, the importance of ERNs in relation to transnational networks was again frequently mentioned:

… the Programme should contribute to the upscaling of networking through ERNs and other transnational networks [33]. (p. 107/7, no. 33; p. 107/28, no. 9d)

However, in practice, no European reference network has been established other than the one addressing rare diseases. Apparently, in practice, it is not that easy for the EU to go transnational when it comes to curative health.

### EU policies for public health: constructing a transnational knowledge strategy while lacking authority

Although the EU had, and has, a limited role and limited authority in issues relating to health care, the EU has developed an agenda to address the health of populations. In 1996, the EU introduced eight so-called Community actions in the field of public health, focusing on ‘health promotion, education and training’, ‘cancer’, ‘AIDS and certain other communicable diseases’, ‘drug dependence’, ‘health monitoring’, ‘injury prevention’, ‘rare diseases’ and ‘pollution-related diseases’. These programmes led to annual work programmes with specific objectives and activities and specific EU funding. Mainly through financial support, the EU encouraged Member States to conduct research and studies, to exchange best practices and knowledge, to promote actions and activities and to encourage greater integration of, for example, health promotion and health education. The programmes were reviewed in 1998, and the Commission concluded that–.

… a new health strategy and programme was needed in view of the new Treaty provisions, new challenges and experiences so far [30]. (p. 271/2)

This led, in 2002, to a more integrated approach to public health:

The general objectives of the programme shall be: […] (c) to promote health and prevent disease through addressing health determinants across all policies and activities [30]. (p. 271/6)

Interestingly, this objective did not change over the following decades. The EU policy documents of 2002, 2007, 2014 and 2021 mention the same aim. The overall aims of public health policy, namely prevention and health promotion, have not changed.

In the first EU document in 2002 [30] about prevention and health promotion, attention was paid to diverse *health determinants* (p. 271/2,3, e.g. no. 6,7, 10), *health promotion* (p. 271/6, e.g. no. 41 /art 2.3.a) *and disease prevention* (p. 271/1, e.g. no. 3). The document explicitly mentions classic lifestyle factors that are assumed to contribute to worse health: ‘*nutrition, physical activity, tobacco, alcohol, drugs and other substances and on mental health’* [30] (p. 271/11, no. 3.1). This perspective is mentioned again in the 2007 and 2014 EU policy documents:

Address health determinants to promote and improve physical and mental health, creating supportive environments for healthy lifestyles and preventing disease; key factors such as nutrition and physical activity and sexual health, and on addiction-related determinants such as tobacco, alcohol, illegal drugs and pharmaceuticals used improperly [31]. (p. 301/11, no. 2.2.1)

The above quote refers to a specific action mentioned in the programme that shall complement, support and add value to the policies of Member States. In line with this, nutrition, alcohol, passive smoking, unhealthy dietary habits and physical inactivity [32] (p. 86/11, no. 1.1) are described as risk factors for health. In the last EU policy document of 2021, a broad spectrum of health determinants is mentioned again:

… such as behaviour-related, biological, socio-economic and environmental factors [33] (p. 107/12, article 2.11).

Our analysis of the policy documents shows that that the EU repeatedly mentions the importance of prevention and health promotion and aims to stimulate knowledge exchange and policy developments between member states about these matters. However, it had no authority to develop policies itself when it comes to prevention and health promotion.

### EU policies for public health in border regions: a selective transnational knowledge strategy while lacking authority

Thus far, we have sketched how the EU has tried to do justice to the notion that, in many respects, promoting the health of EU citizens requires transnational work, while at the same time being bound by the principle that health matters are a national and not an EU concern. In this context, how do EU policy discourses address public health in border regions?

In the 2007 EU policy document, referring to specific health threats, the EU highlighted the importance of considering health issues from a transnational perspective and of organising European cross-border collaboration:

The European Parliament and the Council of the European Union, acting in accordance with the procedure laid down in Article 251 of the Treaty (3), Whereas: A number of serious cross-border health threats with a possible worldwide dimension exist and new ones are emerging which require further Community action. The Community should treat serious cross-border health threats as a matter of priority. The Programme should place emphasis on strengthening the Community’s overall capacities by further developing cooperation between the Member States [31]. (p. 301/3, no. 5)

Due to the serious nature of cross-border threats to health, the Programme should support coordinated public health measures at Union level to address different aspects of such threats [33]. (p. 107/3, no. 11)

The above policy directives led to the general objective in 2021 of ‘*protecting people in the Union from serious cross-border threats to health and strengthening the responsiveness of health systems and coordination among the Member States in order to cope with serious cross-border threats to health*’ [33] (p. 107/12).

In these policy documents ‘cross-border’ is used primarily in the context of a ‘threat’, but what is considered a threat? The EU policy documents of 2002, 2007 and 2014 mention a variety of biological and chemical threats– such as E. coli, influenza strain H1N1, SARS or toxic chemicals. The first document of 2002 particularly emphasises communicable diseases as a threat:

… developing an information system for the early warning, detection and surveillance of health threats, both on communicable diseases, including with regard to the danger of cross-border spread of diseases (including resistant pathogens), and on non-communicable diseases [30]. (p. 271/10, no. 12)

In the 2014 document, the notion of cross-border health threats was broadened to include environmental events and hazards due to climate change. The document specifically refers to the EU decision of 2013 on serious cross-border threats to health:

Apart from communicable diseases, a number of other sources of danger to health, in particular related to other biological or chemical agents or environmental events, which include hazards related to climate change, could by reason of their scale or severity, endanger the health of citizens in the entire Union, lead to the malfunctioning of critical sectors of society and the economy and jeopardise an individual Member State’s capacity to react [32]. (p. 293/1)

In order to minimise the public health consequences of cross-border threats to health as set out in Decision No 1082/2013/EU of the European Parliament and of the Council (1), which could range from mass contamination caused by chemical incidents to pandemics, like those unleashed recently by E. coli, influenza strain H1N1 or SARS (severe acute respiratory syndrome), or health effects resulting from increasing population movements, the Programme should contribute to the creation and maintenance of robust mechanisms and tools to detect, assess and manage major cross-border health threats [32]. (p. 86/3, no. 15)

Protect Union citizens from serious cross-border health threats: 2.3 Actions required by, or contributing to, the implementation of Union legislation in the fields of communicable diseases and other health threats, including those caused by biological and chemical incidents, environment and climate change [32]. (p. 86/11)

In the 2021 EU policy document, the notion of a ‘cross-border threat’ was again raised, and this document explicitly argues for EU efforts to address these disasters:

… ‘border threat to health’ means a life-threatening or otherwise serious hazard to health of biological, chemical, environmental or unknown origin which spreads or entails a significant risk of spreading across the national borders of Member States, and which may necessitate coordination at Union level in order to ensure a high level of human health protection [33] (p. 107/12, no. 9)

The risks of cross-border biological, chemical and environmental threats introduces a rationale to argue for EU policy and EU authority, but apparently, this EU policy discourse was not extended to include prevention and health promotion. While one can observe in EU policy documents the development of a perspective on social health problems, social health problems are not been considered from a European, cross-border perspective. In the EU policy documents, alcohol consumption, drugs and tobacco are not constructed as urgent cross-border problems, and these documents do not argue for European, transnational efforts. EU policy documents that were analysed associate ‘cross-border’ only to ‘threats’ in the sense of ‘a life-threatening or otherwise serious hazard to health’ [33] (p. 107/12, no. 9), whereby these threats result in acute health emergencies and disasters.

## Discussion

Informed by insights from constructivist policy discourses, we analysed how public health in border regions was addressed and constructed in EU policy. What kind of story is narrated through EU policy discourse? As the EU has no formal authority over health issues, what can be observed is that the EU has carefully navigated the carving out of niches where it may address health issues from an EU perspective. Our analysis shows that EU health policy discourse increasingly acknowledges the importance of transnational perspectives and practices, but that no attention is given to the specific characteristics of border regions in relation to public health.

In the field of border studies, scholars have studied the multiple meanings of borders, beyond borders as geopolitical and historical demarcations. Everyday life in border regions testifies to the fluidity of borders and to the cross-border mobility and engagement of citizens living in border regions. For these citizens, the centre of the nation state is sometimes more distant than that of the neighbouring country. However, it appears to be difficult for EU policies to relate to that reality. The document analysis shows that European policies are transnational by definition [44], but European policies do not necessarily consider border regions in Europa where state-borders are practically non-existent in everyday life [45], even when the term ‘cross-border’ is used in EU policy discourses. Our analysis furthermore shows that even when EU policies address prevention and health promotion as a European phenomenon, practices in border regions are not taken into account. Whether this is a problem is a normative question: citizens in border regions have the freedom to tinker with diverse national governance regimes, benefitting from these different governance regimes or to experience the drawbacks when national policies, as during COVID-19 prevent them from their normal mobility.

Barry [20–22] has argued that transnational knowledge controversies are an important vehicle in the construction of transnational policies and practices. From this perspective, it is not surprising that the EU has invested in a European knowledge base in the health domain to extend national knowledge infrastructures. The analysis indicates that, though it lacks authority in the domain of health care because it is a national responsibility, the EU regards transnational knowledge infrastructures as a route to stimulate transnational governance. There are many other examples where the EU mediates transitional knowledge infrastructures, but in the policy documents we analysed, the focus is on governance bodies like the ECDC, the EMA and ERNs as constituting knowledge infrastructures.

Resonating with the work of Kingdon [23] and the initiatives of Kohll [8] and Decker [9], the recent outbreak of the COVID-19 pandemic has created ‘windows of opportunity’ that allowed the EU to place the transnational perspective on health higher on the agenda. Following Bacchi [24–27] in our analysis of policy discourses, we observed that EU policy discourse defines cross-border health issues in a rather selective way, namely as biological, chemical and environmental problems. This problem definition constructs health problems in such a way that they can be solved by already existing infrastructures in public health and disaster control. This problem construction demonstrates the difficulty of creating new directions. Cross border socio-economic health problems that are also related to smoking, alcohol consumption and poverty, are excluded from the European agenda. To date there have been no ‘windows of opportunity’ to prioritise prevention and health promotion in border regions in EU policies.

Our analysis does not address recent debates about the Europeanisation of health, but is does speak to it. While the concept Europeanisation has several different meanings, most scholars point to the impact of European structures on a national level. Radaelli [46] defines Europeanisation like: ‘ Processes of (a) construction, (b) diffusion, and (c) institutionalization of formal and informal rules, procedures, policy paradigms, styles, ‘ways of doing things’, and shared beliefs and norms which are first defined and consolidated in the making of EU public policy and politics and then incorporated in the logic of domestic discourse, identities, political structures, and public policies.’ In the context of this debate, Martinsen [47] has argued that studies of the Europeanisation of health that repeatedly show limited Europeanisation in the field of health care are mostly performed in Western European countries with old, national bureaucracies. Interestingly, public health is not analysed in these studies, while public health problems– disasters– are dealt with transnationally. Moreover, our analysis shows the importance of knowledge and technologies as vehicles of transnational governance, which raises the question of what can be learned about the Europeanisation of health if integration of knowledge and technologies and the making of European standards (e.g. perinatal death) were to be systematically studied. Knowledge infrastructures and standards may be considered ‘soft tools’ compared to legally binding policies when it comes to Europeanisation, but ‘soft tools’ may have a massive practical impact. From that perspective, it is also interesting to study specific knowledge infrastructures and collaborations for public health in border regions that developed in the absence of EU policies for that goal.

## Limitations

We are aware that not all local, regional and national policies relating to prevention and health promotion in border regions are reflected in EU policy documents. However, this does not alter the fact that we did include the four most important policy documents in this study. These four policy documents form the basis for the actions that the EU has taken and will take in relation to healthcare in Europe in the continuous period from 2002 to 2027. We presented the selected policy documents to EU experts who confirmed our selection. In doing so, we reduced the limitation as far as possible.

It would be interesting to know more about the background to the emergence of the analysed documents. For example, the analysis of policy documents did not include the national regimes and laws of different member states. This could have an impact on why, to date, the EU has no real authority on cross-border prevention and health promotion. However, since EU policy was developed in coordination with all Member States that belonged to the EU at that time, these four policy documents show that this is the direction the Member States could all agree on at that time.

## Conclusion

To conclude, our analysis of policy documents reveals that, while the EU respects the authority of the Member States in health matters, it is also committed to developing a European transnational health policy to address the challenges and limitations of national approaches. Our analysis also indicates that the EU has been able to translate this transnational commitment into a policy agenda and a knowledge strategy and into limited authority with respect to health care. Although the EU also developed a European policy agenda and a knowledge strategy for public health, prevention and health promotion, it was not able to construct authority in these matters. When it comes to threats resulting in (potentially) acute health emergencies and disasters on EU level, border regions are considered as an object for EU policy, taking advantage of the European Health Union, but when it comes to the broader field of public health in border regions, a European mindset or European knowledge strategy has not yet been developed. European Union policies for public health, prevention and health promotion are based on the collaboration of Member States and do not take the everyday realities of border regions into account.

## Data Availability

The dataset generated and analyzed during the current study is available in the European Union repository, https://eur-lex.europa.eu/homepage.html.

## Abbreviations

ECDC: European Centre for Disease Control
ECJ: European Court of Justice
EMA: European Medicines Agency
ERN: European reference network
EU: European Union
OECD: Organisation for Economic Co-operation and Development
WHO: World Health Organisation
